# Effects of Cholesterol Levels on Mortality in Patients with Long-Term Peritoneal Dialysis Based on Residual Renal Function

**DOI:** 10.3390/nu10030300

**Published:** 2018-03-03

**Authors:** Yen-Chung Lin, Yi-Chun Lin, Chiung-Chi Peng, Kuan-Chou Chen, Hsi-Hsien Chen, Te-Chao Fang, Shian-Ying Sung, Mai-Szu Wu

**Affiliations:** 1 Graduate Institute of Clinical Medicine, College of Medicine, Taipei Medical University, 111 Taipei, Taiwan; yclin0229@tmu.edu.tw (Y.-C.L.); misspeng@tmu.edu.tw (C.-C.P.); Kuanchou@tmu.edu.tw (K.-C.C.); 2 Department of Internal Medicine, School of Medicine, College of Medicine, Taipei Medical University, 111 Taipei, Taiwan; 570713@yahoo.com.tw (H.-H.C.); fangtc@tmu.edu.tw (T.-C.F.); 3 Division of Nephrology, Department of Internal Medicine, Taipei Medical University Hospital, 111 Taipei, Taiwan; 4 Division of Endocrinology & Metabolism, Department of Medicine, Taipei Veterans General Hospital, 112 Taipei, Taiwan; f084533@yahoo.com.tw; 5 Faculty of Medicine, National Yang-Ming University, 112 Taipei, Taiwan; 6 Department of Urology, Taipei Medical University-Shuang Ho Hospital, 235 New Taipei City, Taiwan; 7 The Ph.D. Program for Translational Medicine, College of Medical Science and Technology, Taipei Medical University, 111 Taipei, Taiwan; 8 Division of Nephrology, Department of Internal Medicine, Taipei Medical University-Shuang Ho Hospital, 235 New Taipei City, Taiwan

**Keywords:** peritoneal dialysis, mortality, malnutrition, residual renal function, lipid

## Abstract

The effect of dyslipidemia on peritoneal dialysis (PD) patients based on the presence of residual renal function (RRF; renal creatinine clearance >2 mL/min/1.73 m^2^) is unknown. Data from the Taiwan Renal Registry Data System between 2005 and 2012 were analyzed to estimate the association between dyslipidemia and mortality in PD patients. Long-term PD patients (*n* = 8032) were divided into groups with (RRF; *n* = 2691, 33.5%) and without RRF (non-RRF; *n* = 5341, 66.5%). The primary outcome was three-year mortality, and multivariate Cox regression was used for survival analysis. After stratifying the total cholesterol (TC) level between the first and third years, the hazard ratio for mortality was estimated. In the non-RRF group, TC < 120 mg/dL was associated with independently increased risk of mortality. In the RRF group, low TC was not independently correlated with increased mortality, but TC > 285 mg/dL was associated with increased risk. PD patients with higher level of TC (>200 mg/dL) in both first and third years of dialysis had significantly lower risk of mortality. In this nationwide cohort study, PD patients without RRF who had low TC level had the highest mortality, in contrast to those with RRF. Malnutrition in long-term PD patients without RRF is an important issue to be monitored.

## 1. Introduction

Cardiovascular disease (CVD) mortality is the main cause of death in chronic kidney disease (CKD) patients under dialysis. Dyslipidemia is one of the most important factors correlated with higher CVD in general population and CKD patients [1]. However, the role of dyslipidemia on mortality in subjects receiving renal replacement therapy is unclear [2]. Malnutrition-related hypocholesterolemia often initiates inflammation and increase mortality in peritoneal dialysis (PD) patients [3]. In our study, multiple confounding factors are present in hemodialysis (HD) patients, making the role of hypercholesterolemia more complicated [4]. The lipid profiles of HD and PD patients also differ [5].

In the course of PD therapy, patients experience body weight increases, and therefore, dyslipidemia had become an important clinical issue [6]. However, the association between metabolic syndrome and prognosis in PD patients varied in different studies [7,8]. Wu et al. [9] reported that the triglyceride/high-density lipoprotein (TG/HDL) ratio was associated with an increased risk of all-cause mortality in a study cohort of 1170 patients after three years of follow-up in female patients on maintenance PD. In contrast to famous studies of individuals under HD, treatment with lipid-lowering agents in PD patients was reported to be associated with a reduced risk of all-cause and CVD mortality [10,11], which can be partially explained by the elevation of apolipoprotein B in PD patients by dialysate high glucose contain [12]. However, how the preservation of residual renal function (RRF; renal creatinine clearance >2 mL/min/1.73 m^2^) in PD patients makes a difference is unclear. In addition, all of these studies had small sample size.

Therefore, we used a nationwide data set to estimate the true association between dyslipidemia and mortality in PD patients.

## 2. Materials and Methods

This study was approved by the ethics committee of Taipei Medical University’s institutional review board (project N201506012, issued date 26 May 2016) and was conducted in accordance with the principles of the Declaration of Helsinki. The waiver of informed consent of each subject was approved by the institutional review board of Taipei Medical University.

Every dialysis unit submits quarterly laboratory reports and adequacy indices (Kt/V or total creatinine clearance from peritoneal or native kidneys) to the Taiwan Renal Registry Data System (TWRDS, established in 1987) to comply with the required triennial dialysis therapy accreditation by the Taiwan Society of Nephrology and with the guidelines of the National Kidney Foundation’s Kidney Disease Outcomes Quality Initiative and the Kidney Disease: Improving Global Outcomes (KDIGO) and to obtain national health insurance (NHI)-associated dialysis quality reimbursements. The dialysis expense are fully reimbursed to each patient by the national health insurance (NHI) bureau in Taiwan. The application for maintenance of dialysis certificated is carefully processed and approved by review of the medical records by two senior nephrologists. Detailed information was uploaded to TWRDS for continuous surveillance, including patient’s comorbidities such as myocardial infarction, coronary heart disease; medication for hypertension, anemia management; biochemical data including TC, fasting TG, calcium (Ca), albumin, phosphorus (P), intact parathyroid hormone, alkaline phosphatase, white blood cell (WBC), hematocrit (Hct) and fasting glucose every 3 months. Eighty percent of the biochemical tests were performed in the Union Clinical Laboratory. Enzymatic methods using a Siemens (Munich, Germany) auto analyzer (ADVIA 1800) were performed to determine general chemistry including cholesterol (coefficient of variation: 1.1%).

**Figure 1:**
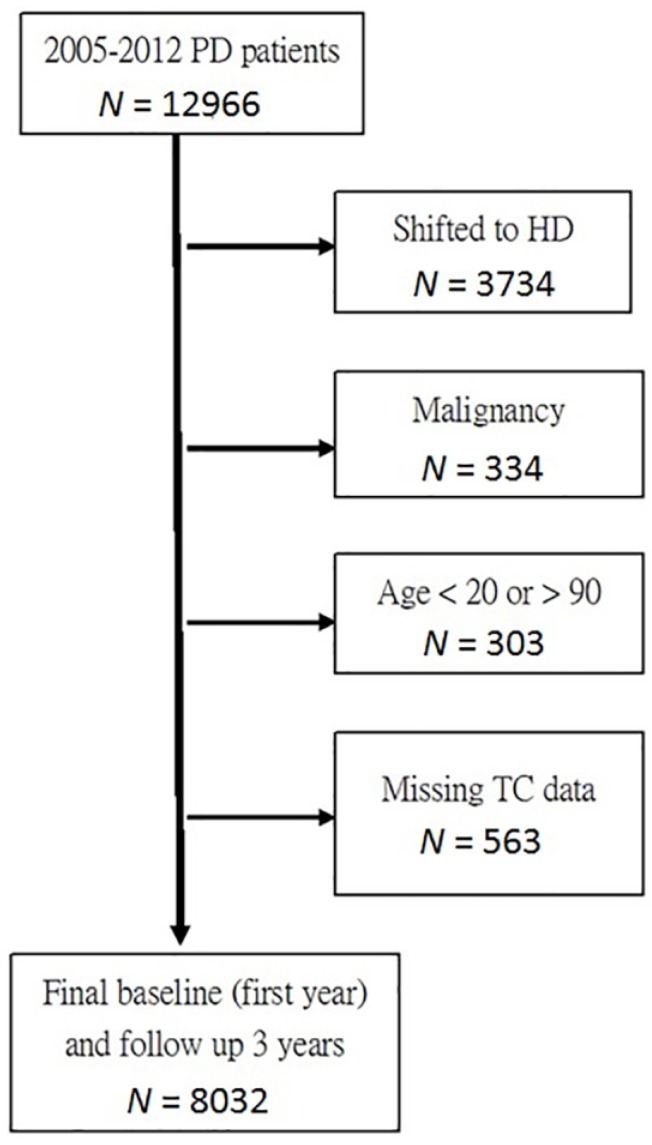
The flow chart of the study population (*n* = 8032). PD: peritoneal dialysis; HD: hemodialysis; TC: total cholesterol.

Patients registered with the TWRDS who underwent long-term PD (>3 months) from 2005 to 2012 were included in the analysis (*n* = 12,966). After excluding patients who had changed their dialysis modality (*n* = 3734), had concurrent malignant tumor (*n* = 334), were age younger than 20 years or older than 90 years (*n* = 303), and had no cholesterol data (*n* = 563), the final sample for analysis included 8032 patients. (Figure 1) 

The primary end point of this study was the 3-year all-cause mortality rate. An individual was regarded dead if he or she was lost to follow-up in the TWRDS based on the complete national coverage provided by the NHI policy for all renal replacement therapy expenditures. Patients were categorized into 14 groups, with an interval of 15 mg/dL by the first year average total cholesterol (TC) level (<120, 120–135, 135–150, 150–165, 165–180, 180–195, 195–210, 210–225, 225–240, 240–255, 255–270, 270–285, 285–300, and >300 mg/dL). We also assessed the effect of change in cholesterol level on mortality during the first 3 years of dialysis. Three groups were created on the basis of total cholesterol (TC) (low: <150 mg/dL, normal: 150–250 mg/dL, high: >250 mg/dL) in the first and third years of dialysis.

All PD patients had weekly kt/V values that met the recommended dose (>1.7 per week). The study group was divided into two groups according to patients’ RRF status. The RRF was defined as renal creatinine clearance (>2 mL/min/1.73 m^2^) according to (Suzuki H. Home Dialysis in Japan, 1st ed.; Karger AG: Basel, 2012; vol. 177, pp. 24–29). It was measured by 24 H urine collection at the first year.

The descriptive statistics were presented as means (standard deviations (SDs)), medians (ranges), or frequencies (percentages) for continuous variables and proportions for categorical variables. The differences between nominal variables were compared by chi-square test, one-way analysis of variance ANOVA or Kruskal Wallis test was used for the analysis of continuous variables, as appropriate, and Log-rank test was used for Kaplan-Meier analysis. Cox regression analysis was performed to estimate the hazard ratio (HR) of 3-year mortality in PD patients for different cholesterol levels. The case mix-adjusted model contained the confounding factors: age, sex, type 2 DM, hypertension, congestive heart failure, left ventricular hypertrophy, history of stroke or acute myocardial infarction, and the use of anti-hypertensive agents. The laboratory data of glucose, Hct, Ca, P, PD treatment adequacy (weekly creatinine clearance), and malnutrition/inflammation markers, including albumin level and White blood cell counts, were also adjusted in the following Cox regression analysis. The level of significance was set at 0.05, two-tailed for all tests. All descriptive and multivariate analyses were performed using the Statistical Package for the Social Sciences software version 17.0 for Windows XP (SPSS Inc., Chicago, IL, USA) and SAS version 9.1 (SAS Institute, Cary, NC, USA).

## 3. Results

### 3.1. Population Demographics

#### Baseline Characteristics of the Two Groups Categorized by Cholesterol Level

Table 1 summarizes the study population’s baseline characteristics categorized by RRF (without RRF, termed non-RRF group: *n* = 5341 HD patients, 66.5%; with RRF, termed RRF group: *n* = 2691 of the whole cohort, 33.5%). The mean (±SD) patient age of the two groups was 55.8 ± 16.9 and 55.4 ± 16.3 years, respectively. Type 2 diabetes mellitus was present in 36% of the whole cohort. The RRF patients had similar mean age, but shorter PD vintage compared with the non-RRF patients; other comorbidities including diabetes mellitus, hypertension, congestive heart failure, left ventricular hypertrophy, and cerebrovascular accidents were similar (Table 1).

### 3.2. Survival Analysis: Kaplan–Meier Survival Curve

**Figure 2:**
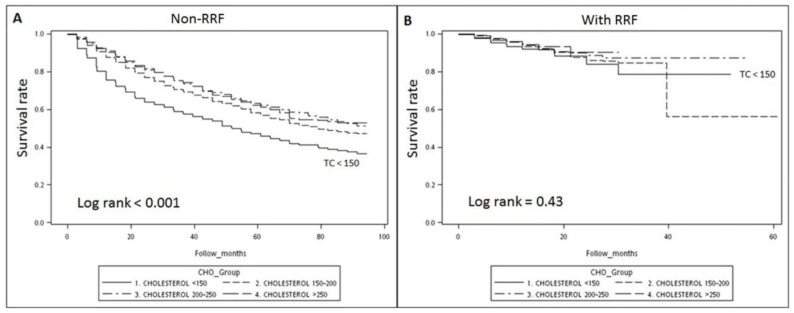
The Kaplan–Meier curve showing that hypocholesterolemia (TC < 150 mg/dL) in patients without RRF (**A**) had the worst survival rate, but not in patients with RRF (**B**).

Figure 2 shows the overall unadjusted Kaplan–Meier survival curve. In the non-RRF patients (Figure 2A), the group with TC < 150 mg/dL had the worst survival rate (log-rank, *p* < 0.001). However, in the RRF patients (Figure 2B), no such effect was observed in the group with TC < 150 mg/dL (log-rank, *p* = 0.43).

### 3.3. Association of Total Cholesterol Levels and Mortality with RRF Stratification

**Figure 3:**
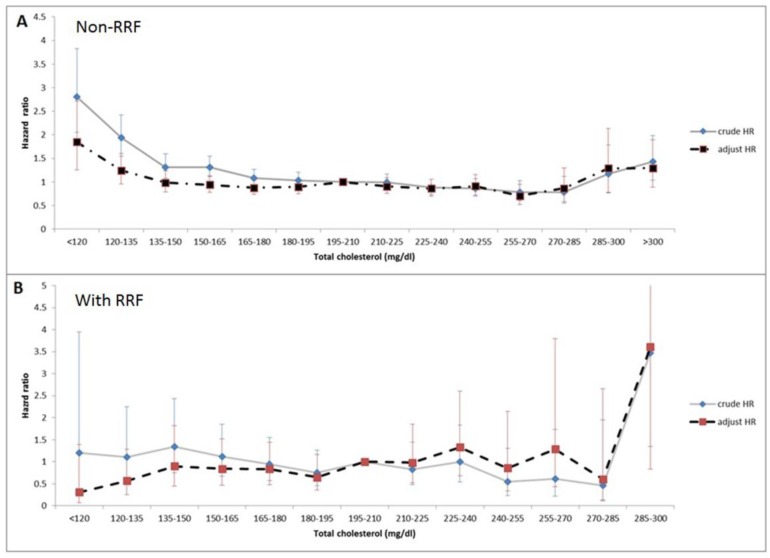
The unadjusted and adjusted hazard ratio (HR) of mortality in short interval TC levels demonstrated an U-shape curve among the patients without RRF (**A**) and linear curve among the patients with RRF (B).

Table 2 shows the crude and adjusted HRs of mortality associated with total cholesterol in the Cox regression model. Case mix and malnutrition/inflammation markers, including WBC counts and albumin levels, were adjusted. In non-RRF group, the adjusted HRs were statistically significant (1.85; 95% confidence interval (CI): 1.26–2.71) in the group with TC < 120 mg/dL, compared with the group with TC of 195–210 mg/dL. In the RRF patients, no increase in HR (0.31, 95% CI: 0.07–1.39) was observed in the group with TC < 120 mg/dL, compared with the reference group with TC of 195–210 mg/dL. Figure 3 shows the curve of the crude and adjusted HRs of TC levels in the non-RRF and RRF groups. A U-shape association between TC and all-cause mortality was observed in the non-RRF group (Figure 3A), whereas a trend of incremental mortality with increasing TC level was observed in the RRF group; however, there was no statistical significance (Figure 3B).

### 3.4. Subgroups Analysis with Stratification by the Existence of RRF

Table 3 shows the subgroups analysis including TC levels, diabetes, hypertension, age, and sex. Presence of type 2 diabetes had a 1.53 times risk of mortality (95% CI: 1.38–1.70) in the non-RRF group. In the contrary, hypertension, younger age, and female sex showed a protective effect on mortality. However, the adverse effect of low TC on mortality was not seen among the RRF group.

Table 4 shows the association of mortality and TC levels in the three groups, and the non-RRF patients with low average TC level in the baseline first year and third year follow-up showed a significantly higher mortality risk (HR: 1.73, 95% CI: 1.50–1.99) compared those with normal TC level (150–200 mg/dL). In addition, the subsequent HR of mortality decreased 35% if their average TC level was normal in the first year and high in the third year. However, in the RRF patients, a similar insignificant effect of low TC levels on mortality was observed (HR: 1.33, 95% CI: 0.86–2.05). A significantly decreasing HR of mortality (71% or 68%, respectively) was detected when the average TC levels increased from normal to high levels or decreased from high to normal in this population. In addition, continuous high TC levels show no statistically protective role in the RRF patients compared with those in the non-RRF patients. (HR 0.85, 95% CI 0.77–0.93).

## 4. Discussion

This is the first nationwide population-based study on the effect of lipid on mortality modulated by RRF on long-term PD patients. A traditional U-shaped mortality was observed when TC is lower than 150 mg/dL and higher than 250 mg/dL in PD patients without RRF. However, PD patients with RRF had a difference pattern, and an increasing trend of mortality was noted when TC levels were increasing, similar to that in the general population [13]. The key point is that low TC level at the beginning of long-term PD therapy is not an ominous sign. Our findings suggest that, at the time of dialysis initiation, it is reasonable to treat dyslipidemia as an extension of lipid-lowering therapy in advanced CKD patients who are not dialysis-dependent, but not in dialysis-dependent patients without RRF. The 2014 KDIGO guideline [14] for lipid management also recommended that statins should not be used in dialysis-dependent patients because the malnutrition-related syndrome may adversely affect mortality [15], but preferring to use statin in all CKD patients with high cardiovascular risks, according the beneficial result of the SHARP study [16].

HD and PD patients may act differently in the first few years of long-term dialysis therapy. First, PD preserves RRF well [17]. Second, PD patients probably may encounter more problems in fluid and weight control, which may be followed by dyslipidemia [18]. Chen et al. [19] described in their longitudinal cohort study that low LDL was correlated with PD patients without RRF and that change in HDL was correlated with faster RRF deterioration. The 4D and AURORA study only focused on HD patients; thus, we believe that the effect of lipid profile on mortality in PD patients with RRF is similar to that in non-dialysis-dependent CKD patients. However, in the course of PD treatment, PD patients with diminished RRF also became susceptible to malnutrition [20], as with HD patients, which is partly the reason why prospective clinical trials on the use of lipid-lowering agents on long-term dialysis patients usually failed in HD patients without RRF [2,21].

Malnutrition is a severe universal problem affecting mortality in dialysis-dependent patients, and in this study, the estimated HR of mortality was 1.73 times if the TC level was normal at the first year and decreased at the third year among long-term PD patients without RRF. In not only dialysis patients but also in advanced CKD patients, low TC level interplayed with malnutrition led to cytokine activation, pro-inflammatory condition, and higher prevalence in CVDs [22]. Although a large double-blind randomized study showed the positive benefit of statin on general population with low cardiovascular risk [23], this correlation is not applicable to dialysis-dependent population, based on the concept of “reverse epidemiology” [24], where lower cholesterol is associated with worsened CVD by itself or maybe confounded by malnutrition or inflammation. Interestingly, a prospective study from Liu et al. [15] reported that in the absence of malnutrition/inflammation, no reverse epidemiology, in terms of the effect of lipid on dialysis patients, was observed. In our study, the markers of malnutrition and inflammation, albumin and WBC counts, were found to be similar between the RRF and non-RRF groups. Even after adjusting for the above parameters, TC level <150 mg/dL still had an increased risk for all-cause mortality in the non-RRF group. This is similar to the observation in people older than 70 years from a study of the Honolulu Heart Program [25], in which the same cohort of patients in the lowest TC group from two examination periods had the worst cardiovascular outcome.

In this study, PD patients with RRF whose TC levels were from high to normal had 66% decreased mortality, which hints that statin treatment may be beneficial. Goldfarb-Rumyantzev et al. [11] demonstrated that incidental PD patients with high TC levels (>226–275 mg/dL) may have survival benefits from statin; albeit, it was a retrospective study. Although, a growing burden of cardiometabolic disease and risk affecting obesity and diabetes may result in hypercholesterolemia and increased mortality, in our study, however, this adverse effect was observed only in the RRF group with TC levels higher than 285 mg/dL , but not in the non-RRF group.

This study had several limitations. First, there was no detailed information regarding anti-platelet, anti-hypertension, and, most importantly, lipid-lowering medication such as statin prescription. The lower TC level may have been due to the effect of medication. Second, the actual cause of mortality, such as cardiovascular origins, cannot be validated from the TWRDS data set. Third, a complete plasma lipoprotein profile in this data set was lacking.

## 5. Conclusions

This nationwide population-based cohort study demonstrated that the effect of lipid profiles on mortality was modified by RRF. Maintaining lipid profiles at normal values is correlated with better survival. In addition, malnutrition, presenting as low TC levels in non-RRF long-term PD patients is a severe clinical problem. Further large prospective randomized studies including therapeutic approaches for those with high TC levels in the RRF group are warranted.

## Figures and Tables

**Table 1:** Baseline characteristics of 8032 PD patients follow up three years by cholesterol.

Variable	Whole Group	Non-RRF	With RRF	*p* Value
Number	8032	5341	2691	
Age (years)	53.3 ± 14.8	53.1 ± 14.8	53.9 ± 14.6	0.014
Male (%)	3702 (46%)	2365 (44%)	1337 (50%)	<0.001
DM (%)	2918 (36%)	1872 (35%)	1046 (39%)	0.001
HTN (%)	3893 (48%)	2692 (50%)	1201 (45%)	<0.001
CHF (%)	656 (8%)	526 (10%)	129 (5%)	<0.001
LVH (%)	578 (7%)	492 (9%)	86 (3%)	<0.001
CVA (%)	269 (3%)	217 (4%)	52 (2%)	<0.001
HTN drugs (%)	6548 (82%)	4402 (82%)	2146 (80%)	0.004
PD duration (years) (25th–75th (median))	(0.79–1.06 (0.91))	(0.82–1.31 (0.94))	(0.64–0.95 (0.84))	<0.001
Following duration (years)	2.11 ± 1.07	2.58 ± 0.88	1.17 ± 0.76	<0.001
**Laboratory data**				
WBC (x1000/uL)	7.22 ± 2.27	7.30 ± 2.34	7.08 ± 2.12	<0.001
nPCR (gm/KgBW/day)	1.04 ± 0.24	1.04 ± 0.24	1.04 ± 0.24	0.23
Triglyceride (mg/dL)	169.5 ± 110.0	174.7 ± 113.1	159.1 ± 102.8	<0.001
Glucose (mg/dL)	125.8 ± 53.9	126.8 ± 56.6	123.7 ± 48.1	0.01
Albumin (g/dL)	3.69 ± 0.45	3.70 ± 0.45	3.65 ± 0.45	<0.001
Hct (%)	30.72 ± 3.67	30.48 ± 3.73	31.19 ± 3.48	<0.001
Ca (mg/dL)	9.09 ± 0.76	9.17 ± 0.77	8.93 ± 0.72	<0.001
P (mg/dL)	5.05 ± 1.13	5.02 ± 1.13	5.11 ± 1.12	0.001
ALK-P (u/L)	118.0 ± 98.9	121.4 ± 100.5	111.2 ± 95.2	<0.001
i-PTH (pg/mL)	269.4 ± 206.6	261.3 ± 206.3	285.3 ± 206.1	<0.001
Ca*P	45.75 ± 11.12	45.89 ± 11.39	45.47 ± 10.56	0.11

RRF: residual renal function; DM: type 2 diabetes mellitus; HTN: hypertension; CHF: congestive heart failure; LVH: left ventricular hypertrophy; CVA: cerebral vascular accident; Hct: hematocrit; Ca: calcium; P: phosphate; ALK-P: alkaline phosphatase; i-PTH: intact parathyroid hormone; WBC: white blood cell.

**Table 2:** Association with total cholesterol and mortality in stratification of RRF.

	Non-RRF (*n* = 5341)			With RRF (*n* = 2691)		
	*n* (%)	Crude HR (95% CI)	Adjust HR (95% CI)	*n* (%)	Crude HR (95% CI)	Adjust HR (95% CI)
Cholesterol (mg/dL)						
<120	65 (1.2%)	2.81 (2.01–3.83) **	1.85 (1.26–2.71) *	45 (1.7%)	1.21 (0.37-3.95)	0.31 (0.07–1.59)
120–135	164 (3.1%)	1.94 (1.55–2.43) **	1.24 (0.96–1.60)	111 (4.1%)	1.10 (0.54–2.25)	0.56 (0.25–1.29)
135–150	302 (5.7%)	1.32 (1.08–1.60) *	0.99 (0.79–1.23)	178 (6.6%)	1.35 (0.74–2.43)	0.90 (0.45–1.82)
150–165	533 (10.0%)	1.31 (1.12–1.55) *	0.94 (0.78–1.13)	330 (12.3%)	1.11 (0.67–1.86)	0.84 (0.47–1.53)
165–180	728 (13.6%)	1.09 (0.93–1.27)	0.88 (0.74–1.04)	408 (15.2%)	0.95 (0.58–1.55)	0.83 (0.48–1.44)
180–195	801 (15.0%)	1.04 (0.89–1.20)	0.90 (0.76–1.06)	431 (16.0%)	0.76 (0.45–1.26)	0.65 (0.36–1.17)
195–210	848 (15.9%)	Ref.	Ref.	361 (13.4%)	Ref.	Ref.
210–225	709 (13.3%)	1.00 (0.85–1.17)	0.91 (0.76–1.09)	300 (11.1%)	0.83 (0.48–1.45)	0.98 (0.52–1.86)
225–240	475 (8.9%)	0.88 (0.74–1.06)	0.86 (0.70–1.05)	218 (8.1%)	1.00 (0.55–1.83)	1.3 (0.68–2.60)
240–255	311 (5.8%)	0.87 (0.71–1.07)	0.91 (0.72–1.15)	135 (5.0%)	0.54 (0.23–1.30)	0.86 (0.34–2.14)
255–270	189 (3.5%)	0.79 (0.61–1.02)	0.71 (0.52–0.95) *	77 (2.9%)	0.61 (0.22–1.74)	1.29 (0.44–3.80)
270–285	97 (1.8%)	0.78 (0.55–1.12)	0.87 (0.58–1.30)	49 (1.8%)	0.47 (0.11–1.95)	0.61 (0.14–2.66)
285–300	46 (0.9%)	1.17 (0.77–1.79)	1.29 (0.78–2.14)	26 (1.0%)	3.47 (1.35–8.93) *	3.61 (0.83–15.70)
>300	73 (1.4%)	1.43 (1.04–1.98) *	1.30 (0.89–1.90)	22 (0.8%)	0	0

* <0.05, ** <0.01

**Table 3:** Hazard ratio (HR) of mortality in subgroups and stratification according to RRF by cox regression methods.

Group	Non-RRF (*n* = 5,341)			With RRF (*n* = 2691)		
Variables	*n* (%)	Crude HR (95% CI)	Adjusted HR (95% CI)	*n* (%)	Crude HR (95% CI)	Adjusted HR (95% CI)
Cholesterol (mg/dL)						
<150	525 (10%)	1.46 (1.28–1.66) **	1.23 (1.05–1.43) **	328 (12%)	1.22 (0.81–1.85)	0.83 (0.52–1.35)
150–200	2329 (44%)	Ref.	Ref.	1289 (48%)	Ref.	Ref.
200–250	1986 (37%)	0.85 (0.78–0.94) **	0.98 (0.88–1.09)	869 (32%)	0.88 (0.64–1.02)	1.28 (0.89–1.84)
>250	501 (9%)	0.86 (0.74–1.00) *	1.03 (0.86–1.23)	205 (8%)	0.79 (0.43–1.43)	1.16 (0.57–2.36)
**DM**						
Y	1872 (35%)	1.82 (1.68–1.98) **	1.53 (1.38–1.70) **	1046 (39%)	1.85 (1.37–2.38)	1.35 (0.96–1.90)
N	3469 (65%)	Ref.	Ref.	1645 (61%)	Ref.	Ref.
**HTN**						
Y	2692 (50%)	0.53 (0.48–0.57) **	0.77 (0.70–0.86) **	1201 (45%)	0.91 (0.69–1.20)	1.10 (0.78–1.55)
N	2649 (50%)	Ref.	Ref.	1490 (55%)	Ref.	Ref.
**Age**						
≤50	2342 (44%)	0.50 (0.46–0.54) **	0.71 (0.64–0.80) **	1075 (40%)	0.51 (0.38–0.70) **	0.81 (0.55–1.19)
>50	2999 (56%)	Ref.	Ref.	1616 (60%)	Ref.	Ref.
**Sex**						
Male	2365 (44%)	Ref.	Ref.	1337 (50%)	Ref.	Ref.
Female	2976 (56%)	0.84 (0.77–0.91) **	0.81 (0.73–0.90) **	1354 (50%)	0.87 (0.66–1.14)	0.71 (0.50–0.99) *

* *p* < 0.05, ** *p* < 0.01, controlling age, sex, diabetes, hypertension, congestive heart failure, left ventricular hypertrophy, cerebral vascular accident, myocardial infarction, use of anti-hypertensive agents, albumin, hematocrit, calcium, phosphate, intact parathyroid hormone, kt/V, White blood cell, and albumin 3.5. Association of mortality and the three different TC groups (low, normal, high) between the first- and third-year follow-up.

**Table 4:** Association of Morality and TC groups (low, normal, high) between the 1st and 3rd year follow-up.

**Non-RRF (*n* = 5341)**	**The 3rd Year**			
	**Low**	**Normal**	**High**	
The 1st Year				
Low	1.73 (1.50–1.99) **	0.55 (0.38–0.78) **		–
Normal	0.89 (0.66–1.21)	Ref.		0.65 (0.52–0.81) **
High	–	0.65 (0.55–0.77) **		0.85 (0.77–0.93) **
**With RRF (*n* = 2691)**	**The 3rd Year**			
	**Low**	**Normal**		**High**
The 1st Year				
Low	1.33 (0.86–2.05)	0.36 (0.09–1.48)		–
Normal	0.44 (0.11–1.76)	Ref.		0.29 (0.09–0.92) *
High	–	0.34 (0.14–0.83) *		0.89 (0.65–1.21)

* *p* < 0.05, ** *p* < 0.01; low: TC < 150 mg/dL; normal: TC 150–200 mg/dL; high: TC > 200 mg/dL.
